# Membrane Thickness as a Key Factor Contributing to the Activation of Osmosensors and Essential Ras Signaling Pathways

**DOI:** 10.3389/fcell.2018.00076

**Published:** 2018-07-24

**Authors:** B. Eleazar Cohen

**Affiliations:** Division of External Activities, National Institute of Allergy and Infectious Diseases, Bethesda, MD, United States

**Keywords:** osmosensors, lipid bilayer thickness, membrane thinning, Ras proteins, signaling pathways, antimicrobial peptides, colistin, amphotericin B

## Abstract

The cell membrane provides a functional link between the external environment and the replicating DNA genome by using ligand-gated receptors and chemical signals to activate signaling transduction pathways. However, increasing evidence has also indicated that the phospholipid bilayer itself by altering various physical parameters serves as a sensor that regulate membrane proteins in a specific manner. Changes in thickness and/or curvature of the membrane have been shown to be induced by mechanical forces and transmitted through the transmembrane helices of several types of mechanosensitive (MS) ion channels underlying functions such as osmoregulation in bacteria and sensory processing in mammalian cells. This review focus on recent protein functional and structural data indicating that the activation of bacterial and yeast osmosensors is consistent with thickness-induced tilting changes of the transmembrane domains of these proteins. Membrane thinning in combination with curvature changes may also lead to the lateral transfer of the small lipid-anchored GTPases Ras1 and H-Ras out of lipid rafts for clustering and signaling. The modulation of signaling pathways by amphiphilic peptides and the membrane-active antibiotics colistin and Amphotericin B is also discussed.

## Introduction

The emergence of the cell as a functional unity enclosed in a semipermeable lipid bilayer is often associated with the existence of a higher osmotic pressure at the cell interior than at the external environment due to the presence of impermeant intracellular metabolites. This gradient of osmotic pressure is one of the most important mechanical forces acting on cell membranes, as it exerts a lateral tension that stretches the lipid bilayer and produce a thickness reduction (Anishkin et al., 2014). Such changes in bilayer thickness sometimes combined with alterations of membrane curvature are now recognized as critical modulators of the function of some membrane proteins contained within them (Andersen and Koeppe, 2007; Lundbaek et al., 2010; Battle et al., 2015). Thus, bacterial MscL channels under mechanical stretch allows unselective leakage of small solutes that prevent cell lysis (Sukharev et al., 1997). In fact, when MscL channels are incorporated into lipid bilayers with shorter lipid hydrocarbon chains, the ion channels gate at a lower membrane tension whereas the closed state is stabilized by increased thickness (Perozo et al., 2002). Osmotic manipulation of membrane thickness produces gating changes in voltage-dependent K^+^ channels (Schmidt et al., 2012) and also enhance the light-induced currents across mechanosensitive transient receptor potential (TRP) and TRP-like (TRPL) ion channels (Hardie and Franze, 2012). X-Ray diffraction studies of membrane-embedded crystals of the Ca^2+^ pump ATPase have recently revealed that variations in membrane thickness of about 2.5 Å allow large tilting movements of the protein TM helices during the reaction cycle (Norimatsu et al., 2017). This finding has firmly established the importance of local thinning effects on the functioning of membrane proteins (Andersen and Koeppe, 2007).

Osmotic forces have also been demonstrated to activate ion channels such as those formed by the antifungal polyene antibiotic Amphotericin B (AmB) (Ruckwardt et al., 1998). This is due to the rigid 24 Å length structure of the AmB molecule that is unable to span lipid bilayers with a thickness greater than the length of the AmB molecule (Cohen, 2016). However, upon a hypo-osmotic stress, a relatively small decrease of 3 Å in the bilayer thickness (from 46 to 43 Å) of sterol-free liposomes prepared with dieicosenyl phosphatidylcholine (DEPC) or dioleoyl-palmitoyl PC (POPC) led a substantial increase of ion channel activity (Ruckwardt et al., 1998). Of note, the threshold of membrane thickness for the appearance of the long-lived AmB aqueous pores in planar lipid bilayers occurred when such bilayers are prepared with cholesterol and C17 chain length phospholipids, which are about 3.6 Å thinner than C18 PC bilayers (Shatursky et al., 2014). Interestingly, it has been estimated that when the McsL bacterial channels are in the open state the lipid bilayer thins by about 5 Ẵ (Elmore and Dougherty, 2003), a reduction in thickness that is somewhat greater that needed to open the long-lived AmB ion channels (Shatursky et al., 2014).

As it is highlighted here, membrane thickness is also a key factor in the activation of bacterial and yeast osmosensors. In addition, the membrane localization of the yeast Ras1 and the mammalian H-Ras into specific lipid nanodomains for downstream signaling appears to depend not only on GTP-binding but also on lipid forces generated by changes on membrane thickness and curvature.

## The activation of signal transduction pathways by osmosensors

Signal transduction in bacteria and plants is often mediated by “two components” signaling system that is formed by a histidine protein kinase (HK) and a response regulator (RR) (West and Stock, 2001). The HKs are membrane proteins that have sensor domains for detecting environmental signals and transmitting them to catalytic cytoplasmic domains. A typical HK has a sensing domain at the periplasmic region flanked by two transmembrane (TM) helices, which are followed by a cytoplasmic region containing both kinase and receiver domains (Mascher et al., 2006). Two TM helices from each monomer of bacterial HKs form a four-helical bundle in the membrane (Mascher et al., 2006). There are several three-dimensional structures of conserved soluble domains of HKs, but no direct structural information is yet available about the TM domains that link the external and the cytoplasmic domains, leaving the mechanism of signal transduction unresolved (Gao and Stock, 2009).

Recently, a model of PhoQ, a bacterial HK that senses high concentrations of divalent cations and/or low pH as part of a two-component PhoQ/PhoP signaling system has been constructed by combining disulfide crosslinking data with homologous crystal structures (Molnar et al., 2014). Thus, by using Bayesian inference methods, Molnar et al., obtained data for two interconverting structures that represents the two functional states, kinase and phosphatase, of the PhoQ sensor. Such interconverting structures mainly differ in the diagonal displacements of the helices within the four TM bundle of the homodimer (Molnar et al., 2014). In this so-called “Scissor Model” of signal transduction, tilting of the TM domains of PhoQ appears to be associated to changes in the cytoplasmic domain bundle which is in an inactive phosphatase conformation when tightly packed, but it is loosely packed in the activated kinase state (Bhate et al., 2015). On the other hand, the X-ray structural evidence that is available for the isolated PhoQ external sensor has indicated that this domain locked the overall membrane structure in the unphosphorylated phosphatase state in the presence of high external concentrations of divalent cations (Cho et al., 2006). Such cations serve as metal bridges between an acidic patch in the sensor and negatively charged lipids at the cell membrane (Cho et al., 2006). These investigators have suggested that depletion of divalent cations from such bridges triggers transmembrane signaling by increasing the repulsion between negative charges close to the external surface (Cho et al., 2006). Similar movements are proposed to be exerted by antimicrobial peptides (AMPs) that compete for overlapping metal binding sites at the membrane surface of PhoQ (Bader et al., 2005).

However, AMPs are also known to activate the PhoQ sensor possibly acting as the actual signal *in vivo* (Rosenberger et al., 2004; Yadavalli et al., 2016). In this regard, it is important to note that binding of LL37 and other cationic AMPs such as colistin at the membrane surface also produce changes in various membrane parameters including thinning of the bilayer (Henzler-Wildman et al., 2004; Mecke et al., 2005; Grage et al., 2016). In addition, it is well-known that divalent cations act by screening negative charges in LPS monolayer membranes producing a more ordered and slightly thicker structures, before their disruptive action on the outer membrane takes place (Clifton et al., 2015). In the absence of divalent cations, a thinner monolayer is observed, a finding that reflects a higher tilt of the hydrocarbon chains relative to the surface normal due to increased repulsion between negatively charged headgroups (Clifton et al., 2015).

**Figure 1:**
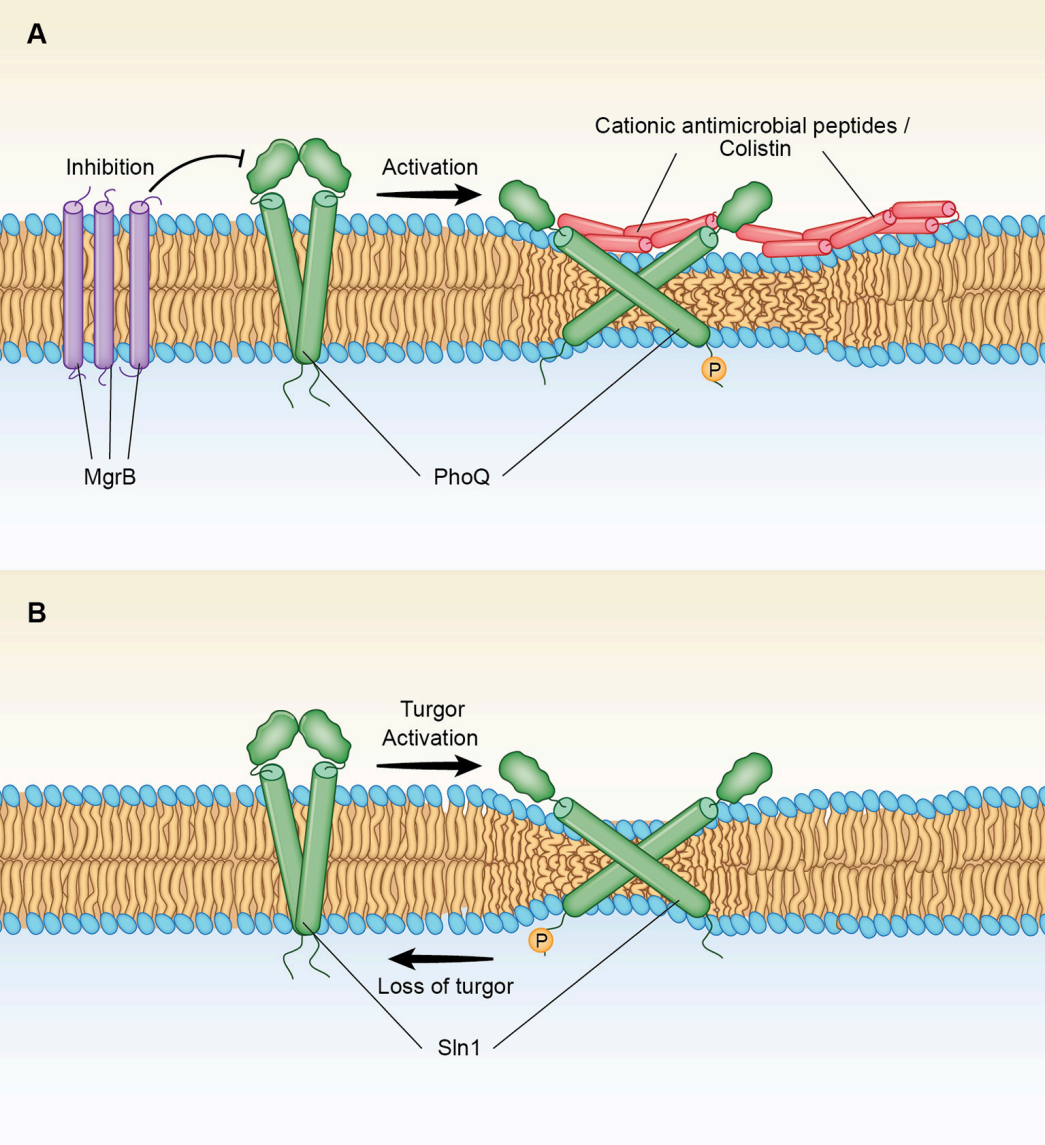
**(A)** Mechanism of activation of the two-component PhoQ/PhoP signaling system by AMPs and colistin. Antimicrobial peptides (AMPs) produce a membrane thinning effect that leads to changes in tilting and displacements of the TMs of PhoQ, a bacterial histidine kinase (HK). Such changes convert the HK phosphatase stage, in which the kinase is deactivated, into a phosphorylated state in which the kinase is activated. MgrB is a small transmembrane protein that is part of the PhoQ/PhoP regulatory circuit. MgrB contains a long hydrophobic domain of 20 aminoacids spanning the membrane that upon oxidation of external cysteine residues (see text) may form thicker aggregates that block PhoQ activation. PhoQ activation. Inactivation of mgrB in Gram-negative bacteria results in Colistin resistance through activation of PhoQ/PhoP (see text). **(B)** Mechanism of activation of HOG by loss of turgor. Sln1 is a histidine kinase (HK) that serves as the osmosensor of the SLN1 branch of the high osmolarity glycerol (HOG) pathway. Increased turgor pressure produces a membrane thinning effect that leads to changes in tilting and displacements of the TMs of Sln1. Such changes convert the phosphorylated state in which the kinase is activated to a phosphatase stage, in which the kinase is deactivated. Accumulation of unphosphorylated Sln1 (and downstream Ssk1) leads to the activation of the HOG pathway.

A similar thinning effect can also be exerted by cationic AMPs or colistin, prior to the disruption of the inner bacterial membrane, forcing a higher tilt of the TM domains of PhoQ that activate this signaling system (Figure 1A). Such a mechanism of activation for PhoQ would be consistent with the observation that the PhoQ regulatory circuit includes a negative feedback involving mgrB, a gene that encodes a protein that blocks the sensor activation (Cannatelli et al., 2014). Thus, MgrB is a small 47-amino acid protein that resides in the inner membrane of the bacteria interacting directly with PhoQ (Lippa and Goulian, 2009). MgrB in S. enterica contains three cysteine residues (amino acids C16. C28 and C39) and a hydrophobic domain of 18 amino acids (MKKFRWVVLGIVVVVCLLLWAQVFNIMCDQDVQFFSGICAINKFIPW) (underlined residues 6–24). Lippa et al., has found that the periplasmic protein DsbA facilitates the oxidation of the MgrB cysteine residues blocking PhoQ/PhoP signaling activity (Lippa and Goulian, 2009). Therefore, it is likely that the DsbA-induced Cys-S-S-Cys connectivity of MgrB stimulates aggregation of the peptide in the membrane (Figure 1A). Such an effect, would promote a “thickness clamp” effect that blocks the AMPs-induced thinning effect on the bilayer, locking the sensor to the un-phosphorylated conformation.

Interestingly, the predicted TM2 domain of PhoQ in S. enterica (residues 193 to 216 WFVYVLAANLLLVIPLLWIAAWW) is flanked at both the cytoplasmic and at the periplasmic side with two consecutive aromatic residues. This preferential location of aromatic Trp and Phe residues at the membrane/water interphase has been predicted to allow large movements of the TM domain perpendicular to the membrane by acting as a membrane “float” that minimizes energy costs (Killian and von Heijne, 2000). In fact, the X-ray studies of the Ca^2+^ pump ATPase have indicated that Trp residues -in cooperation with basic Arg amino acid residues at the interface–allow changes in the thickness of the bilayer during the protein reaction cycle by changing their interactions with membrane phospholipids and in this way reducing the energetic costs (Norimatsu et al., 2017).

## Mechanism of activation of the HOG pathway via the SLN1 osmosensor

The high osmolarity glycerol (HOG) signaling pathway in fungal cells is known to be activated under a hyperosmotic shock by the rapid loss of cell volume due to water efflux (Schaber et al., 2010). However, the mechanism of such activation remains unclear. The HOG pathway is made of the SLN1 and SHO1 branches, which contain membrane proteins that serve as osmosensors (Reiser et al., 2003; Saito and Posas, 2012). Reiser et al. have reported that nystatin in the absence of any osmotic stress activated the SLN1 pathway, by inducing the leakage of intracellular ions via the aqueous pores formed, an event that leads to water efflux and the reduction of cell volume. The Sln1 osmosensor of the HOG pathway is a HK that has sequence similarities to the two-component bacterial and plant HKs (Reiser et al., 2003). Like other bacterial HK sensors, the fungal Sln1 has a sensing domain at the periplasmic region flanked by two transmembrane (TM) helices, which are followed by a cytoplasmic region containing both kinase and receiver domains (Mascher et al., 2006). At external osmolarities compatible with normal cell growth, the Sln1 kinase is auto-phosphorylated but at high external osmolarities it is de-auto phosphorylated transmitting the signal through the Sln1–Ypd1–Ssk1 multistep phosphorelay to the redundant pair of kinases Ssk2 and Ssk22 (Horie et al., 2008; Saito and Posas, 2012). Thus, under conditions leading to loss of turgor, unphosphorylated Sln1 and Ssk1 will accumulate activating the Hog1 kinase (Horie et al., 2008).

The “Scissors Model” of PhoQ activation can also be extended to the fungal Sln1 with turgor loss acting as the activation signal (Figure 1B). Thus, the model predicts that a reversal of the membrane thinning induced by a hyperosmotic stress will allow the perpendicular movement of the TM domains of Sln1 back toward the membrane normal, converting the phosphorylated state in which the kinase is activated to a phosphatase, a stage that leads to the downstream activation of MAP kinases of the HOG1 pathway (Saito and Posas, 2012). Regarding such TM movements, it is noted that there is a Try residue at the cytoplasmic boundary of the predicted TM2 domain of Sln1 in S. cerevisiae (underlined residues 334–356 KLAKIITGTVIAIGVFVILLTLPLAHW), but the two hydrophobic Ile residues that are present at the periplasmic side, are followed by a segment containing two positively charged Lys residues (KLAK). These positively charged aminoacids upstream of TM2 may be interacting with negatively groups of membrane phospholipids, potentially playing a role in modulating membrane thickness as observed for the TMs of the Ca^2+^-ATPase (Norimatsu et al., 2017). Of importance, a recent *in vivo* cross-linking and MD simulation study of EnvZ, a major osmosensor HK in *E. coli*, has demonstrated a change in signal output caused by repositioning the aromatic residues flanking the TM2 domain (Yusuf et al., 2017). This outcome was found to be correlated to tilting rather than to a vertical displacement of the TM2 helix (Yusuf et al., 2017), a finding that clearly supports a diagonal scissoring mechanism (Falke, 2014; Molnar et al., 2014) for HKs that sense osmotic membrane stretch.

The “Scissor Model” of PhoQ activation, as proposed here for Sln1 activation (Figure 1B) is also consistent with the results of the functional analysis performed by Reiser et al., swapping domains between Sln1 and Cre1, a HK that serves as a cytokinin receptor in plants but it is also sensitive to osmotic changes (Reiser et al., 2003). Thus, these investigators demonstrated that the periplasmic domains of Sln1 and Cre1 are interchangeable with no effect on function in spite of the significant differences between their primary structures. On the other hand, the hybrid HK constructs with swapped TM domains of Sln1 and Cre1 were not activated under an osmotic stress, a clear indication that a particular TM–cytoplasmic domain combination is unique for each histidine kinase (Reiser et al., 2003). The importance for HK activation of each TM-cytoplasmic combination is illustrated by the contrary effects that TM tilting may have on the kinase autophosphorylation at the cytoplasmic domain of PhoQ (Figure 1A) as compared to Sln1 (Figure 1B). Of note, global analysis of the cytoplasmic HAMP domains (named for being present in Histidine kinases, Adenylate cyclases, Methyltransferases, and Phosphodiesterases) has indicated that helical tilts in this domain are a large component of the input-output signaling in HKs (Bhate et al., 2015, 2018).

## Mechanism of activation the SHO1 branch of the HOG pathway

In yeasts, there is a second branch of the HOG pathway that is activated by the Sho1 osmosensor (Tatebayashi et al., 2015). Sho1 is a four-TM domain protein that serves as a scaffolding for the Cdc42 GTPase, the Ste20 PAK-like kinase, the Ste11 kinase, the Ste50 adaptor and the Pbs2 kinase (Tatebayashi et al., 2015). Of importance, Sho1 is localized in lipid rafts (Tanigawa et al., 2012), which are signaling platform microdomains present in the membranes of eukaryotic organisms (Simons and Sampaio, 2011; Mollinedo, 2012; Zhou and Hancock, 2015) as well as in bacteria (Toledo et al., 2018). Lipid rafts are considered equivalent and with similar biophysical properties to the liquid-ordered (L_0_) domains that are formed in model lipid membranes (Hancock, 2006). Lipid rafts are also called nanoclusters to reflect the observation that in biological membranes they are formed on very short time and length scales as compared to those characterized in model membranes (Zhou and Hancock, 2015).

The formation of lipid rafts in biological membranes appears to depend on the self-assembly properties of sphingolipids and sterols (Klose et al., 2010; Simons and Sampaio, 2011). In fact, it has been recognized that the tilting degree of sterols in phospholipid membranes is critical for the formation of such structures (Aittoniemi et al., 2006) as well as for the stability and clustering of proteins such as Sho1 (Tanigawa et al., 2012) and H-Ras (Lin et al., 2015). Of importance, under turgor loss, there is an increased clustering of the Sho1 osmosensor within the lipid rafts (Tanigawa et al., 2012), an effect that enhance the interaction of the four TM domains of Sho1 with Ste50 (Tatebayashi et al., 2015). Therefore, the HOG pathway activation can be directly related to the conformational changes in the Sho1 TM domains that are induced by turgor loss. This mechanistic model would be consistent with the finding that in the absence of any osmostress, the expression of a Sho1-P120L mutant in yeasts activates the HOG pathway (Tatebayashi et al., 2007). Thus, the mutated Pro120 residue of S. cerevisae Sho1 is in the middle of the second external loop upstream of two lysines that precede the predicted TM4 helix (underlined residues 124 to 143 G**P**KKAAASAGVILLSIINLIWILYY). In the Sho1-P120L mutant, the substitution of Pro by a hydrophobic Leu amino acid residue may enhance the interaction of any of the two positively charged lysines with the negatively lipid head groups at the membrane surface. Consequently, the TM4 helix of Sho1 may be forced to tilt with respect to the membrane normal, as it is expected to occur upon the reversal of the membrane thinning induced by the loss of turgor. Similar displacements of a TM domain by a positively charged-lipid anchor at the membrane surface has been demonstrated to play a role in the activation of mechanosensitive bacterial ion channels (Bavi et al., 2017). In the case of Sho1, the initial vertical displacement of the TM4 domain may in turn displace the rest of TMs that are closely interacting with each other (Tatebayashi et al., 2015).

Additional meaningful information provided by other Sho1 mutant constructs is also consistent with an activation model of the HOG pathway based on osmotically-induced thickness changes in the membrane. Thus, substitution with Ala of amino acids W139 and I140 at the end of the TM4 helix, blocks not only the binding of Sho1 with Ste50 but also the activation of the HOG pathway via the HKR sub-branch (Tatebayashi et al., 2015). In fact, Hkr1 is a single TM protein that is involved in signaling by interacting with Sho1 and the Ste20 kinase (see model in Figure 4 of Tatebayashi et al., 2015). Upon a hyperosmotic stress, the single TM domain of Hrk1 may be vertically displaced by changes in membrane thickness leading to the activation of Ste11 by the Ste20. This is an event that appears to precede the Sho1/Ste50-mediated activation of Pbs2 and Hog1 (Tatebayashi et al., 2015). In this regard, the observed inhibition by nystatin of the SHO1 branch of the HOG pathway (Reiser et al., 2003) has been explained as due to the blocking of the initial Hrk1/Ste20 interaction (Tatebayashi et al., 2015). This effect is not related to the osmotic changes induced by the aqueous pores formed by nystatin as it was not observed in the presence of natamycin, a polyene antibiotic that is unable to form ion channels (Te Welscher et al., 2008). It is then likely that the sterol/polyene complexes themselves formed by both antibiotics at the external membrane leaflet can exert a curvature effect blocking the vertical displacement of the Hrk1 TM domain that precedes the activation of Ste11 by Ste20.

## Membrane-based activation of ras proteins

Ras are small GTPases that are found in most eukaryotic cells acting as molecular switches of MAPK pathways, promoting cell growth and other related functions in coordination with environmental factors (Chen and Thorner, 2007; Gutin et al., 2015). All Ras proteins have in the N-terminal region a highly-conserved G-domain that contains the GDP/GTP binding site and switch loops I and II where the interactions for activators and effectors takes place (Hancock, 2003). At the C-terminal end, Ras proteins display a hypervariable region (HVR) that includes the motif CAAX with a Cys residue, which is irreversible farnesylated (Hancock, 2003; Tamanoi, 2011). The C-terminal end of fungal Ras and mammalian Ras (except for K-Ras isoforms) (Table 1) also have 1 or 2 conserved Cys residues where a unique reversible palmitoylation takes place (Linder and Deschenes, 2003; Tamanoi, 2011). In the case of H-Ras, two cysteines (Cys181 and Cys184) are palmitoylated whereas N-Ras is palmitoylated only at the extra Cys181 (Hancock, 2003). The loss of the farnesylation in Ras renders all the isoforms cytosolic as they are unable to target any membrane (Hancock, 2003). On the other hand, palmitoylation increases the overall Ras affinity for the plasma membranes with the palmitoylation/depalmitoylation cycle regulating its spatial distribution between the cell membrane and endomembranes (Hancock, 2003; Onken et al., 2006; Grecco et al., 2011).

**Table 1:**
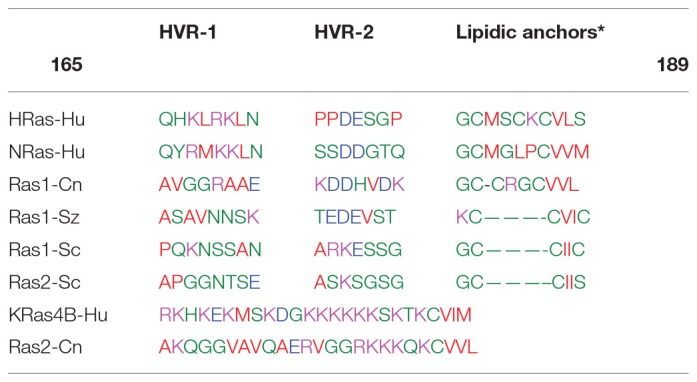
Amino acid sequence alignments of the C-terminal hypervariable region (HVR) in different Ras proteins isoforms.

*The differences in amino acid sequences between mammalian Ras proteins (HRas-Hu, NRas-Hu, KRas4B-Hu) and fungal Ras1/Ras2 proteins are mainly in their C-terminal hypervariable region (HVR). Numbers at the two sides of H-Ras C-terminal indicate amino acid positions. Hu, Human; Cn, Cryptococcus neoformans; Sz, Schizosaccharomyces pombe; Sc, Saccharomyces cerevisiae*.

^*^The HVR-1, HVR-2 and Lipid anchor domains at the C-terminal end of H-Ras are named following Rotblat et al. (2004).

The fully lipidated Ras proteins are inserted in the plasma membranes at transient nanoclusters (Zhou and Hancock, 2015). In all the mammalian Ras isoforms, the entire C-terminal region including the HVR domain is known to play a critical role in determining their lateral segregation and functional localization into specific nanoclusters (Eisenberg and Henis, 2008). Thus, the H-Ras-associated nanoclusters exhibit a distinct composition of anionic lipids that appear to determine its HVR domain making specific interactions with the membrane phospholipids (Zhou and Hancock, 2015). Notably, a GTP-loaded H-RasG12V (HΔhvrG12V) construct without the HVR-1 domain (residues 165–179) (Table 1) resides entirely in the cholesterol-rich lipid rafts with its lateral segregation and subsequent activation of effectors blocked (Rotblat et al., 2004). More specifically, MD simulations of the GDP-bound H-Ras have indicated that the basic amino acids R169 and K170 in the flexible HVR-1 linker (Table 1), interact with the membrane (Gorfe et al., 2007b). MD simulations and cell -based mutagenesis studies have also revealed that the G-domain of the GTP-bound H-Ras form changes its orientation from perpendicular to parallel to the membrane surface, allowing basic R128 and R135 residues at helix α4 to be in direct contact with the membrane phospholipids (Gorfe et al., 2007b; Abankwa et al., 2008; Guzmán et al., 2014). These experimental data combined with the computer simulations support a model in which the GDP-bound H-Ras form, is distributed between the cholesterol-sterol-rich lipid rafts and sterol-poor membrane areas but upon GTP binding H-Ras resides predominantly in more disordered areas (Prior et al., 2001). However, there is not direct experimental evidence demonstrating that the lateral transfer of H-Ras out of the lipid rafts is triggered solely by GTP binding.

## The effect of changes in cell volume on H-ras activation

It has been recognized for some time that in mammalian cells opposite patterns of cell function including growth are triggered by hypo-osmotic alterations of external osmolarity as compared to hyperosmotic changes (Schliess et al., 2007). In effect, in response to a hypo-osmotic change, human intestinal cells exhibited an increased activity of the H-Ras/Raf1/Erk pathway (Van der Wijk et al., 1998). However, the physiological relevance of these changes is unclear because no osmosensors have been characterized in mammalian cells and contrary to fungal organisms, in all mammals the extracellular aqueous environment is under homeostatic regulation. In this respect, it was recently reported that during mitosis, HeLa cells in suspension as well as adherent cells exhibited an increase from 10% up to 30% in cell volume (Son et al., 2015; Zlotek-Zlotkiewicz, 2015). Son et al., have also demonstrated that the observed increase in cell volume during mitosis in such cells was inhibited by blocking the Na-H exchanger (Son et al., 2015), a system that is known to regulate the increase in volume after hypertonic shrinkage (Alexander and Grinstein, 2006). Notably, the Na-H exchanger is activated by the H-Ras/Raf1/MEK pathway (Hooley et al., 1996).

The activation of Ras-mediated signaling pathways in mammalian cells is well-known to be associated with the binding of ligands into receptor tyrosine kinases (e.g., growth factor receptors, T cell receptors) (Groves and Kuriyan, 2010). This activation process involves recruitment into the membrane of SOS (Son of Sevenless), a GEF that converts the inactive GDP-Ras to active GTP-Ras, playing a key role in signal integration (Gureasko et al., 2008; Christensen et al., 2016). However, recent work on K-Ras4B has revealed that GDP/GTP exchange may not be sufficient for downstream effector activation from the plasma membrane (Jang et al., 2016; Nussinov et al., 2018). In the case of the H-Ras isoform, to complete the activation process, GTP-loaded H-Ras may need to be transferred out of the lipid rafts driven by the membrane thinning induced by cell swelling. Interestingly, in the case of the GDP-bound N-Ras, an isoform that is known to be located at sterol-poor disordered domains (Henis et al., 2009), it was found that the activation volumes for binding at high hydrostatic pressure into a pure synthetic fluid membrane yielded overall positive reaction volumes (ΔV_R_ > 0) (Kapoor et al., 2013). Such positive values are indicative that upon membrane binding there is a local thinning by lateral expansion (Kapoor et al., 2013).

## An activation mechanism of fungal ras proteins by changes in membrane thickness

In fungal cells, a turgor pressure of 0.8–1.5 bar appears to be strong enough to cause a small but significant thinning of the cell membrane (Schaber et al., 2010; Chang, 2017). This condition is often associated with the growth stage in fungi (Basu et al., 2014; Chang, 2017). In fact, upon turgor loss, yeast cells initiate a complex set of responses that include a temporary delay of the cell-cycle progression possibly to prevent any interference with the transcription of adaptive genes to hyperosmotic stress (Saito and Posas, 2012).

Several studies have demonstrated that there is a functional interchangeability between the yeast Ras1 and mammalian H-Ras (DeFeo-Jones et al., 1985; Bond et al., 2013), a finding indicative of the conservation of similar membrane activation mechanisms, possibly including lateral transfer into distinct nanoclusters upon activation. In this regard, it is known that the contribution of amino acids R169 and K170 at the HVR-1 domain of H-Ras to the lateral segregation requires that this region to be correctly spaced relative to the location of the membrane lipidic anchors (Rotblat et al., 2004). In *S. pombe* and *S. cerevisiae*, the Ras1 isoforms have the same spacing than in H-Ras between the HVR-1 domain and the first palmitoylated Cys (Table 1). However, in both fungal isoforms, the equivalent positively charged residues R169 and K170 in HVR-1 are replaced by non-charged amino acids asparagine or serine/threonine (N or S/T in Table 1). Such changes may facilitate the lateral displacement out of the ergosterol-rich lipid rafts of the signaling-competent Ras1-GTP form as it was found to be case for H-Ras after replacements of R169 and K170 residues by alanines (Abankwa et al., 2008).

**Figure 2:**
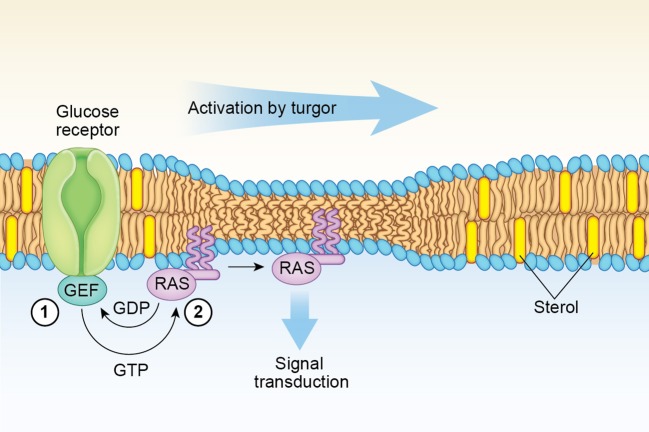
Model for fungal Ras1 activation by Turgor. In the step labeled 1, glucose-binding by a G-protein coupled receptor leads to an increase in the GTP-bound Ras1 concentration at the lipid raft boundary mediated by a guanine exchange factor (GEF) such as Cdc25. In step 2, increased turgor exerts a membrane thinning effect that induces lateral transfer and clustering of GTP-bound Ras1 monomers out of lipid raft boundaries driven by a more favorable entropic environment. A similar lateral membrane transfer mechanism induced by cell swelling is proposed for H-Ras (see text).

Of note, the acyl chain composition of the sphingolipid-rich lipid rafts in yeasts is unusual in that they contain very long saturated C26:0 fatty acids (Dickson et al., 2006). Such long saturated acyl chain sphingolipids, which are predominantly located in the external leaflet, can interact with PS and other anionic lipids species that are mostly concentrated in the membrane inner leaflet, facilitating the stabilization of highly curved membrane domains (Schneiter et al., 2004). In this respect, the location in Ras1 of the farnesyl and palmitate anchors adjacent to each other (Table 1) may determine its partitioning at the boundary of lipid rafts, as their positioning at this interface decreases the line tension that exist between the lipid rafts and the surrounding membrane (García-Sáez et al., 2007; Weise et al., 2010; Larsen et al., 2015). It is then likely that under turgor, changes in the balance of forces between membrane thickness and curvature at the boundaries of lipid rafts may be critical to determine the lateral segregation of Ras1 into sterol-poor regions of the membrane where increased clustering and signaling takes place (Eisenberg and Henis, 2008). Thus, turgor-induced membrane thinning may increase the line tension at the raft interface as there is a greater difference in thickness between lipid raft and non-raft domains (García-Sáez et al., 2007). In response to such unfavorable energetic cost, the microdomain sizes could not only change but the farnesyl and palmitoyl chains anchoring the Ras 1 protein are transferred from the curved interface to a more planar area of the membrane (Figure 2). Molecular dynamics (MD) computer simulations studies have indicated that membrane thinning leads to a deeper penetration of the lipidic anchors of H-Ras into the sterol-poor areas of the membrane (Gorfe et al., 2007a). Such MD simulations has also revealed that a deeper penetration into the membrane of a hydrophobic portion of an anchored protein leads to an increased clustering due to the entropy loss, which is caused by local conformation restrictions of the phospholipid hydrocarbon chains at the two bilayer leaflets (Li et al., 2010).

As in the case of H-Ras discussed above, the completion of the lateral transfer of Ras1 out of the sterol-rich nanoclusters to initiate the signaling cascade requires the presence of an increased concentration of the GTP-form of Ras1 at the interface boundaries (Figure 2). The reason is that GTP-binding is needed to produce the conformational changes that will allow the interaction of basic amino acid residues at helix α4 of the Ras isoform with the membrane surface (Gorfe et al., 2007b; Abankwa et al., 2008). The increase in the GTP-Ras1 form is greatly enhanced by the activation of the Ras GEF, Cdc25, via the glucose-binding G-protein coupled receptor Gpr1 (Rudoni et al., 2001; Belotti et al., 2006; Lorenz et al., 2010). In this way, the concentration of the GTP-bound Ras1 state is dependent on the energetics of the cell and the external glucose concentration (Busti et al., 2010).

## The cross-talk between the HOG and the ras pathways

During osmoregulation, the HOG pathway functions as a central signaling mediator by sharing some protein kinases with two other MAPK pathways, the pheromone pathway and the filamentous pathway (FP) (Saito and Posas, 2012; Brewster and Gustin, 2014). Thus, the Ste11 kinase (MAPKKK) is a component of all the three pathways, which interacts with the osmosensor Sho1 via the Ste50 adaptor (Saito and Posas, 2012; Tatebayashi et al., 2015). In this regard, earlier studies revealed that under hyperosmotic stress, yeast deletion mutants of Hog1 (or Pbs2) exhibited an aberrant phenotype that indicated the activation of the pheromone response and the filamentous growth pathway (Davenport et al., 1999). Such an activation of the otherwise independent pathways, has been proposed to be caused by the hyperstimulation of Ste11 (Brewster and Gustin, 2014).

The FP and the HOG pathways also share the osmosensors Hkr1 and Msb2, which interact with the membrane protein Sho1 (Tatebayashi et al., 2007; Yang et al., 2009). Under normal osmotic conditions, the FG pathway is activated by a mechanism involving the Msb2 osmosensor provided that the cells are grown in a low nutritional medium (Vadaie et al., 2008; Yang et al., 2009). In fact, the expression of Msb2 is enhanced under nutrient-limiting conditions, a factor that appears to determine the activation of the FG pathway (Abdullah and Cullen, 2009). Notably, the Ras2/cAMP//PKA signaling, which is a pathway involved in nutrient regulator, has also been found to be required for FG pathway activation in yeasts (Gimeno et al., 1992). In this way, the lack of fermentable sugars triggers the glucose-sensing Gpr1 and its associated Gpa2 protein that regulate Ras2/cAMP-dependent PKA signaling via the GAPs Ira1 and Ira2 (Cullen and Sprague, 2012).

Studies of the genetic interactions in *S. cerevisiae* under different environmental stresses has also revealed a crosstalk between the HOG pathway, the Ras/cAMP/PKA pathway and a protein complex that is called Mediator, which is known to play a significant role as integrator of transcription factors of signaling cascades (Gutin et al., 2015). By analyzing the inter-pathway interaction data, these investigators have concluded that Ras1, plays a critical role to maintain the repression of stress-esponsive genes (Gutin et al., 2015). This conclusion is supported by the finding that the deletion of Ira2 (ΔIra2) under a hyperosmotic stress led to a decreased HOG activation but Δras1 mutants still exhibited increased Hog1-related responses (Gutin et al., 2015). Notably, deletion from yeast cells of Ras2 (Δras2) or Gpa2 (Δgpa2), which are proteins necessary for glucose-induced cAMP production, does not increase HOG activity under hyperosmotic stress (Gutin et al., 2015). In this regard, the epistasis analysis carried out by these investigators revealed that whereas Δhog1 is epistatic over Δras1, Δras2 is epistatic over Δpbs2, which means that Ras1 is situated upstream of Hog1 in the pathway but Pbs2 is upstream of Ras2 (Gutin et al., 2015). This data is consistent with a model in which both yeast Ras1 (Figure 2) and the HOG osmosensors (Tanigawa et al., 2012) are activated by an osmotically-induced laterally segregation into spatially distinct plasma membrane nanoclusters, with a cross-talk between them occurring at downstream effectors (Gutin et al., 2015). On the other hand, Ras2 appears to be present mainly at endomembranes, a conclusion that is supported by the findings that Ras2 and its effectors are localized in the ER and mitochondrial membranes (Belotti et al., 2012) as well as in the nucleus of cells growing exponentially on glucose medium (Broggi et al., 2013).

A functional specialization and/or distinct localization of the Ras1 and Ras2 isoforms in yeasts is also consistent with the observation that the observed resistance to the oxidative killing of yeast cells by AmB was associated with a greater decrease in the generation of oxidative ROS in mitochondria in Δras1 cells than for Δras2 (Belenky et al., 2013). Thus, these results agree with the observation than under hyperosmotic stress, Δras1 mutants but not Δras2 mutants can maintain the generation of Hog1-induced responses (Gutin et al., 2015), including increased production of catalases that confer ROS-protective effects against AmB action (Cohen, 2016).

A cross-talk between the Ras and MAPK pathways has also been observed in mammalian cells under osmotic stress. Thus, after growth factors activate the corresponding membrane receptors, Vero cell lines under hyperosmotic stress exhibited normal levels of GTP-bound Ras and the mammalian Hog1 homolog p38 is activated (Copp et al., 2005). Notwithstanding, the activation of Ras downstream effectors such as Raf, Akt or Erk1/Erk2 was strongly reduced by a mechanism that involves blocking signaling at pathway components downstream of active Ras (Copp et al., 2005).

## The action of AmB on the lateral segregation of fungal ras isoforms

The formation of aqueous pores by AmB in fungal cell membranes is facilitated when such cells are under turgor, as an increased osmotic pressure leads to a thinner cell membrane (Cohen, 2016). Cationic peptides capable of exerting by themselves membrane thinning effects can then be expected to act synergistically with AmB to kill fungal cells. In fact, this synergy was demonstrated to occur in the case of colistin, an antibacterial effect that by itself does not have any effect on fungal cells (Teixeira-Santos et al., 2016).

Addition of AmB (or nystatin) to fungal cells exerts an opposite effect that turgor by inducing the loss of salt and water produced via the aqueous pores formed (Reiser et al., 2003). Thereafter, however, such aqueous pores remain embedded into a non-stretched cell membrane with a thickness, which is somewhat greater than the length of the AmB/sterol complexes forming the aqueous pores (Cohen, 2016). In those membranes regions where AmB aqueous pores are formed, the local thinning is maintained preventing the transfer of Ras1 into the sterol-rich lipid rafts and keeping the Ras1/Ras2/cAMP/PKA pathway activated and generating responses that contribute significantly to the generation of the toxic ROS species (Belenky et al., 2013; see Figure 2).

## Activation of the cell wall integrity (CWI) pathway by osmosensors

The cell wall integrity (CWI) pathway is another membrane-based signaling mechanism present in fungal cells that is capable to sense turgor pressure (Levin, 2005). Thus, membrane stretch induced by reduction in external osmolarity increases activation of CWI signaling (Kamada et al., 1995). Cell volume increases in yeast mutants defective in K+ transport also exhibited constitutive CWI signaling (Merchan et al., 2004). The CWI signaling pathway is composed of the membrane proteins Wsc and Mid2, which are located upstream of a linear cascade of proteins kinases including Pkc1 and a MAP kinase (Mpk1/Slt2) (Levin, 2005).

Wsc and Mid2 proteins contain a large extracellular o-mannosylated serine-threonine region (STR) that is connected to a single TM domain (Verna et al., 1997). Both domains are probably responsible of detecting mechanical stress in the cell wall or the plasma membrane (Kock et al., 2016). In this respect, increased multimerization of Wsc or Mid2 sensors could be the key factor for signaling activation driven by clustering of the single TM domains of various proteins in lipid rafts (Langosch and Arkin, 2009; Kock et al., 2016). Interestingly, the CWI pathway in yeasts is activated by AmB at a concentration range −0.5–0.8 μM- in which aqueous pores are formed (Straede et al., 2007). Chlorpromazine, the amphipathic molecule that activate MS bacterial channels by causing changes in membrane curvature (Martinac et al., 1990) is also a potent activator of Mpk1 (Kamada et al., 1995).

The activation of the CWI pathway activation by AmB can then be the consequence of the local reduction in the bilayer thickness and/or associated membrane curvature produced by the aqueous pores formed at the boundaries of lipid rafts containing the osmosensors. Such membrane changes may cause a change in the orientation (tilt) of the TM within the bilayer, leading to a conformational change that brings the cytosolic domain closer to form functional multimers that triggering the CWI pathway activation. Consistent with the localization of the CWI pathway sensors in specific lipid rafts, it was found that the CWI pathway is activated by inhibitors of fatty acid synthase (FA), acetyl-CoA carboxylase and myriocin, an inhibitor of sphingolipid synthesis (Straede et al., 2007).

## Cross-talk between the CWI pathway and ras in higher fungi

In the basidiomycetous yeast C. neoformans (Cn), a higher fungal species, there are two Ras isoforms (Cn Ras1 and Cn Ras2) that share overlapping functions, but also play distinct signaling roles (Waugh et al., 2002; Nichols et al., 2009). The lipidic anchors of Cn Ras1 resemble the one found in H-Ras with two palmitoylated cysteines upstream of the terminal farnesylated cysteine (Table 1). It is noted (Table 1) that the HVR-1 domain of Cn Ras1 has an alanine in the position occupied by K169 of H-Ras, a factor that may facilitate the lateral displacement and signal activation out of the sterol-rich lipid rafts (Abankwa et al., 2008). On the other hand, the HVR of Cn Ras2 resembles the mammalian K-Ras4B with a single farnesylated cysteine as lipid anchor and several consecutive positively charged residues (Table 1).

Cn Δras1 mutant cells exhibited a decrease in growth, as expected from the fact that Ras1/Ras2 plays a redundant role in the activation of the cAMP/PKA pathway (Waugh et al., 2002). However, a stronger decrease in cell growth occurred in Cn Δras1 strains that are under a hyperosmotic stress, an indication of the role played in this process by the loss of turgor-induced inactivation of the CWI pathway (Maeng et al., 2010), The importance of the CWI pathway in protecting Cn cells against the toxic effects produced by AmB aqueous pores is also consistent with the finding that Cn cells exposed to a combination of moderate hyperosmotic stress with a very low AmB concentration led to a strong decrease in growth (Maeng et al., 2010).

Regarding the cross-talk interactions between Cn Ras1 and the CWI pathway, Maeng et al., found that Cn Δcdc24 mutants upon hyperosmotic stress, exhibited response phenotypes to oxidative and genotoxic stress that are very similar to those shown by Δras1 (Maeng et al., 2010). Cdc24 is a GEF protein and a downstream effector of Cn Ras1 that mediates thermotolerance and actin cytoskeleton regulation (Nichols et al., 2007). In fact, Cdc24 acts as a GEF for Cdc42, a Rho-like GTPase, which is a central switch in the activation of over 20 effector proteins involved in different cellular functions (Phillips et al., 2008), including those responsible of the membrane-protective effects of the CWI pathway (Maeng et al., 2010) as well as in cytokinesis and the organization of the septin proteins responsible of bud morphogenesis (Ballou et al., 2013). In addition, the Ras1/Cdc24 interaction mediates the activation of the Rho GTPases Rac1/Rac2 that primarily regulate polarized growth in C. neoformans (Ballou et al., 2013).

In mammalian cells, Cdc42 and Rac1 also plays an important role in cell polarization, proliferation and migration (Simi et al., 2015; Ozdemir et al., 2018). Recently, single-molecule analysis of Rac1 mobility performed in migrating fibroblasts has revealed that this small Rho paralog-in a similar way than Ras proteins -forms signaling platforms segregated into active nanoclusters that have a specific lipid composition and are distributed as gradients matching the protein subcellular activity (Remorino et al., 2017). Rac1 and RhoA are differentially required for the proliferation of endothelial vs. smooth muscle cells under mechanical stretch (Liu et al., 2007). It follows that the lack of a cell wall in mammalian cells not only allows a diversity of cell shapes but also determine an increased contribution of the lipid-anchored Rho GTPases in combination with the extracellular matrix and the cytoskeleton to the functions of different cells (Huang and Ingber, 2005; Simi et al., 2015).

## Conclusions

The lipid bilayer is one of the primordial cell structures that can respond to mechanical forces by exerting conformational changes on membrane proteins involved in signaling cascades. Thus, changes in membrane thickness induced by osmotic changes appear to lead to tilting of the TM domains of the fungal HK Sln1 and the scaffold protein Sho1 that transmit a signal via specific downstream MAPK pathways. In this regard, although there is not yet direct structural data, the available evidence strongly supports an important role of diagonal scissoring of the TM domains for signal transmission by this type of sensors (Gushchin and Gordeliy, 2018). On the other hand, the signaling function of Ras1 and H-Ras isoforms appears to depend not only on GTP-binding -as it has been classically considered in all Ras activation/deactivation models- but also on its lateral transfer out of lipid rafts induced by thickness and curvature changes.

The studies described here also raise new questions to investigate such as how essential mechanical parameters are maintained in different cells and how the lipid bilayer-induced forces interplay with the mechanical properties of integrin and adhesins (or fungal cell-wall proteins) and the cytoskeleton to regulate specific functions during cell growth and pathogenesis processes. In addition, the increasing evidence that supports the role of biophysical membrane properties in the localization of the Ras and Rho family members into specific lipid nanodomains makes it possible to define new innovative approaches for improving the therapy of fungal and cancer diseases. Efforts in this direction are driven by the remarkable progress made in the use of multiscale computer simulation approaches to understand protein dynamics and its impact on events that can be propagated over long distances such as signaling via Ras and its downstream effectors (Papaleo, 2015; Lu et al., 2016). However, for a complete understanding of the structural mechanisms underlying these processes, it is essential to solve the X-ray structures of the full-length proteins associated with the lipid membrane.

## Author contributions

The author confirms being the sole contributor of this work and approved it for publication.

### Conflict of interest statement

The author declares that the research was conducted in the absence of any commercial or financial relationships that could be construed as a potential conflict of interest.
